# Supplementary material for: Functional comparison of metabolic networks across species
Source: Nat Commun. 2023 Mar 27;14:1699. doi: 10.1038/s41467-023-37429-5 (PMC10043025; doi:10.1038/s41467-023-37429-5)
Supplement: Supplementary file 1 — Supplementary Information [file 41467_2023_37429_MOESM1_ESM.pdf]

Functional comparison of metabolic networks across species
—Supplementary Information—

Charlotte Ramon[1,2], Jörg Stelling[1,*]

[1] Department of Biosystems Science and Engineering and SIB Swiss Institute of Bioinformatics, ETH Zurich, 4058 Basel, Switzerland
[2] PhD Program Systems Biology, Life Science Zurich Graduate School, Zurich, Switzerland
[*] Corresponding author: joerg.stelling@bsse.ethz.ch

Supplementary Figures

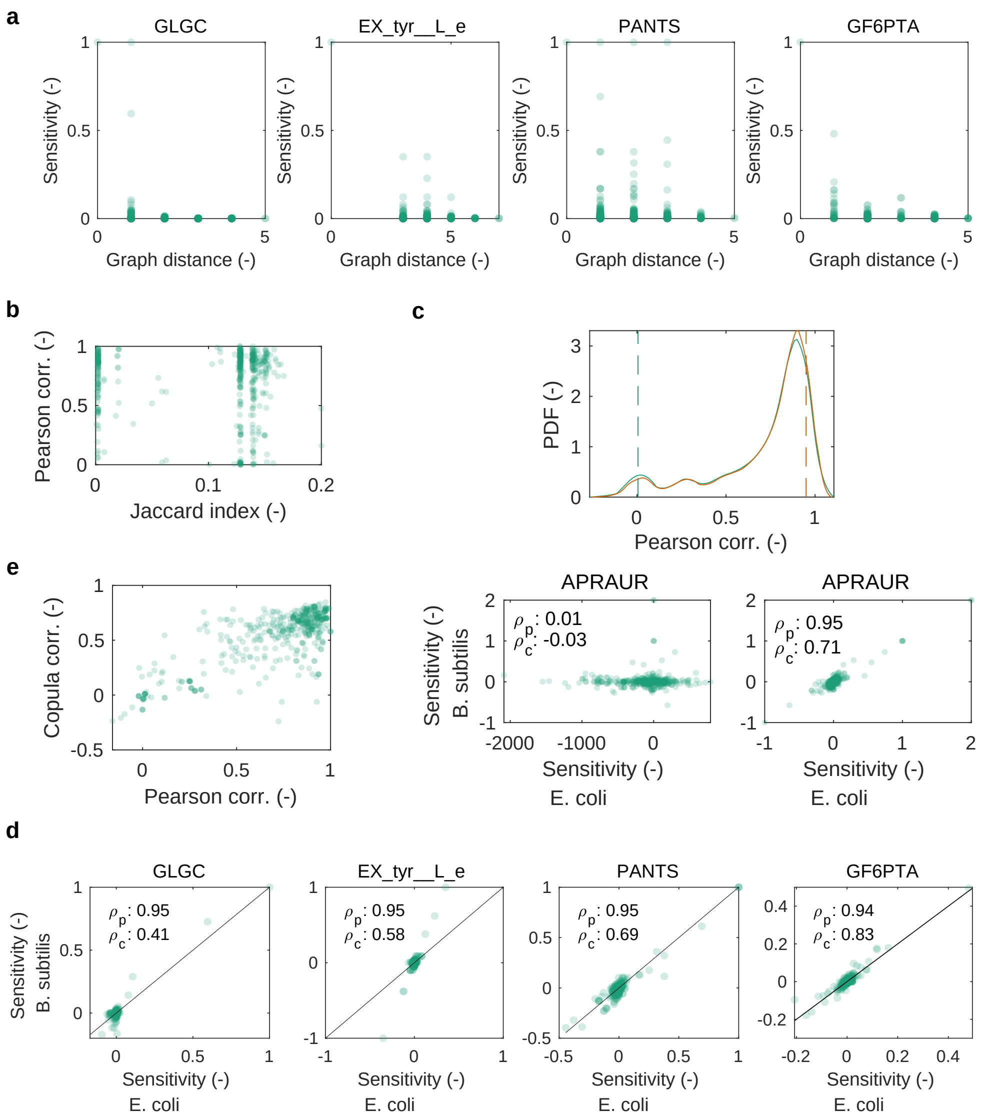

Figure S1. Sensitivities, their relationships to graph distances and specific examples. a Sensitivity as a function of graph distance, as computed using Dijikstra's algorithm, for the *E. coli* model. Selected metabolic reactions: Glucose-1-phosphate adenylyltransferase (GLGC), exchange of L-tyrosine (EX_tyr_L), pantothenate synthase (PANTS) and glutamine-fructose-6-phosphate transaminase (GF6PTA). **b** Comparison of Pearson correlation coefficient and Jaccard index as **Fig. 1b** but for 2-neighborhoods. **c** Top, the green (orange) solid line indicates the kernel density estimate of the Pearson correlation coefficient for common reactions before (after) adding the exchange of riboflavin in the *E. coli* model. The dashed lines indicate the correlation for reaction APRAUR (catalyzed by 5-amino-6-(5-phosphoribosylamino)uracil reductase and involved in riboflavin metabolism) in the corresponding models. Bottom, sensitivities for reaction APRAUR. **d** Sensitivities for the four reactions in *E. coli* and *B. subtilis* with Pearson correlation coefficients equal to 0.95. The diagonal (solid line) represents where the sensitivities should lie if the models were identical. **e** Comparison of Pearson correlation coefficient and copula correlation coefficient for comparison of *E. coli* and *B. subtilis*.

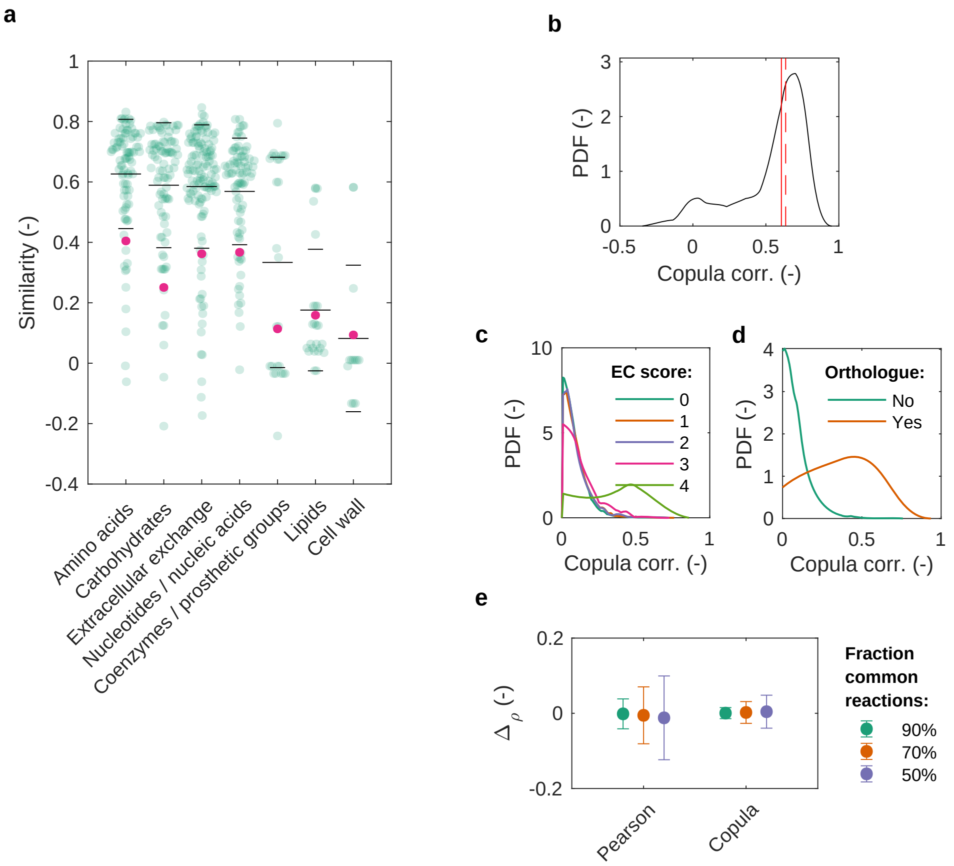

Figure S2. Similarities of metabolic subsystems and different ways to compute their correlations (Pearson, copula). a As **Fig. 2a**, but with copula correlation (green points). **b** As **Fig. 1d**, but with the robust copula correlation coefficient. **c,d** Same as **Fig. 2b,c** but with copula correlation. **e** Influence of the number of common reactions on the measure (either Pearson correlation or copula correlation) for the pair of models *B. subtilis* vs. *E. coli*. A fraction of common reactions was bootstrapped $n = 100$ times for each fraction and the correlation was computed based on the reduced number of common reactions assumed. The averages (points) and standard deviations (error bars) of the differences between the resulting correlation measures and the original measures are represented.

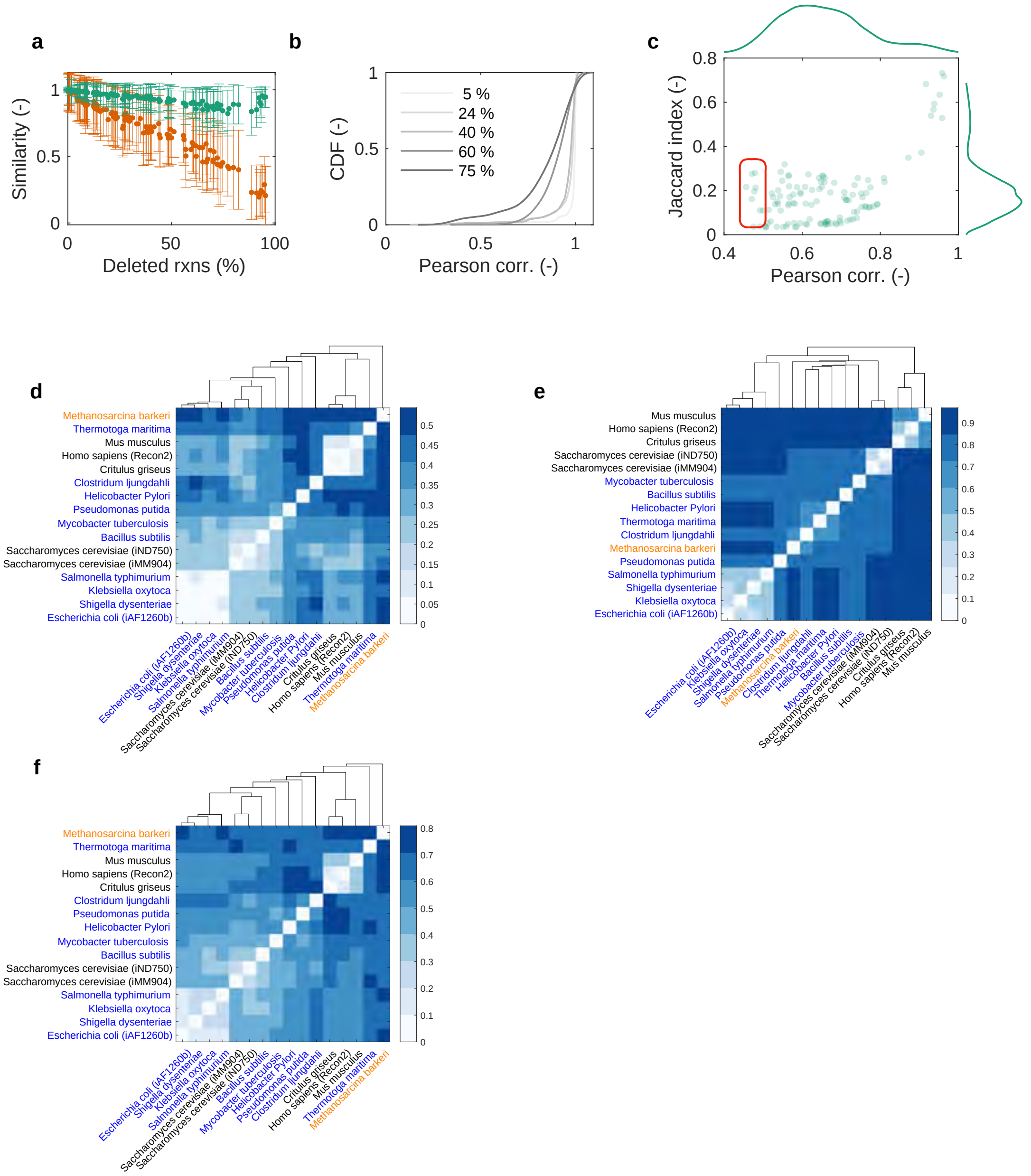

Figure S3. Effects of metabolic repertoire differences on sensitivity correlations and effects of different hierarchical clustering algorithms and measures on phylogenetic analyses. a,b Average similarity of the yeast model with itself, as a function of the fraction of reactions deleted in the second model (x-axis). Averages (points) and standard deviations (error bars) of Pearson correlations (green) and copula correlations (orange) for the remaining common reactions (**a**). Examples of sensitivity correlation cumulative distribution functions (CDF) obtained for different fractions of deleted reactions. Line colors indicate the percentage of deleted reactions in the second model (**b**). **c** Comparison of the Jaccard index and the average Pearson correlation coefficient for each model pair. Points circled in red: model pairs involving *Methanosarcina barkeri*. Solid lines: estimates of the kernel density function. **d,e,f** Dendrograms and associated sensitivity dissimilarity matrices constructed using weighted average distance (WPGMA) (**d**), Jaccard index (**e**) or copula correlation (**f**).

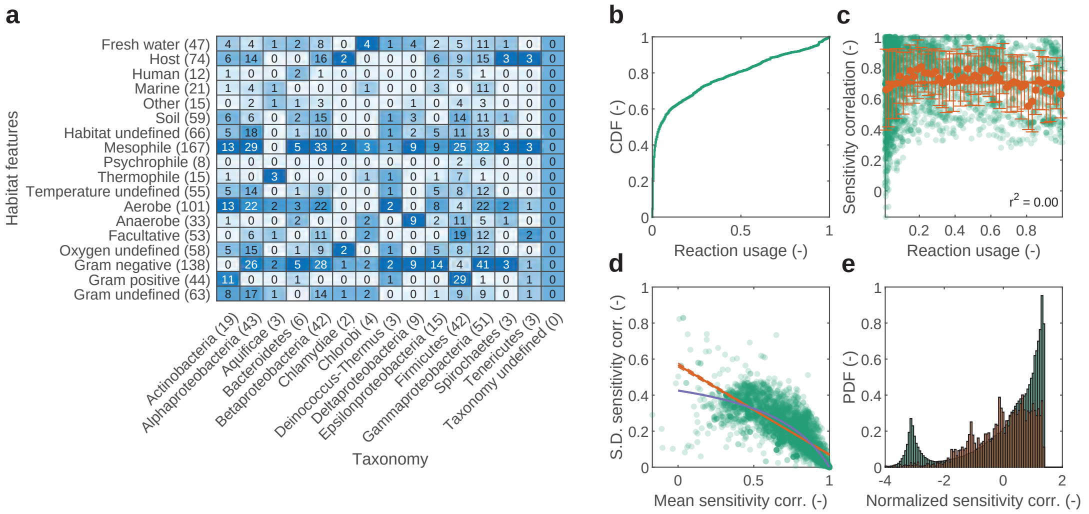

Figure S4. SEED model and sensitivity correlation statistics (Pearson correlation, NCBI taxonomy). a Model features for habitat, physiology, and taxonomy; shading scaled by taxonomy (columns). Habitat categories (top to bottom): habitat, temperature, oxygen, Gram status. Numbers: number of models with corresponding features. **b** Cumulative density function (CDF) of reaction usage, that is, fraction of model pairs a reaction participates in. **c** Normalization of sensitivity correlations by linear regression as a function of reaction usage; reaction usage has no significant impact (Spearman ρ) on sensitivity correlations. Green: individual data (average sensitivity correlations for $n = 3,491$ unique reactions); orange: binned data (mean $\pm$ s.d.) and linear regression. **d** Heteroskedacity of sensitivity correlations per reaction. Green: individual data; red: linear regression; purple: logistic regression. **e** Probability density functions (PDFs) of normalized sensitivity correlations for individual model comparisons (green) and reaction means (orange).

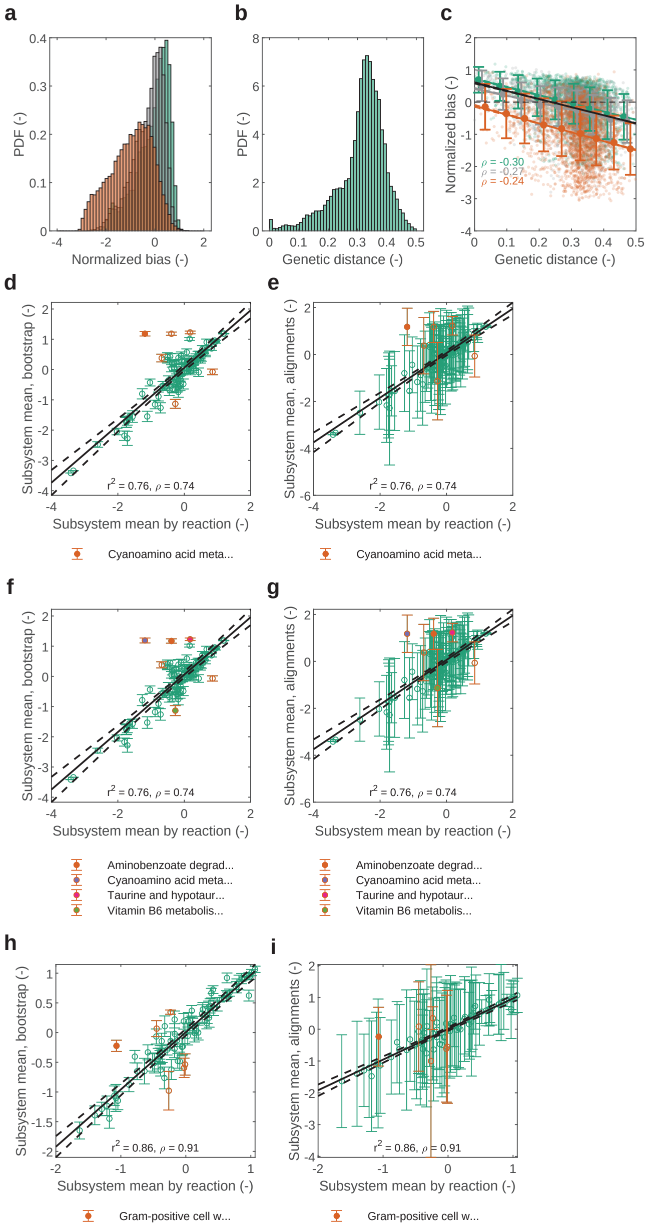

Figure S5. Consistency of subsystem classification (Pearson correlation). **a** Distributions of normalized biases per reaction class in conserved (green), variable (orange) and other (gray) subsystems; $n = 29,483$ average normalized biases per reaction class from pairwise comparisons of the 245 models. **b** Distribution of genetic distances between bacteria (see **Methods** for details). **c** Normalized bias per reaction classified by subsystem type as in (**a**) as a function of genetic distance; biases decrease consistently with genetic distance (Spearman ρ). Small dots: individual data points ($n = 29,483$ per class), large dots and error bars (mean $\pm$ s.d.) of data binned by genetic distance; lines: linear regressions on individual data per class (colors) or for all classes (black). **d,e** KEGG subsystem annotation ($n = 102$ subsystems), subsystem significance based on Wilcoxon signed rank test. Correlations between average normalized biases per reaction and averages obtained by bootstrap of alignments using $n = 100$ bootstrap samples (**d**) or over raw sensitivity correlations over all aligned reactions ($n = 20,210,490$ correlations) (**e**). **f,g** KEGG subsystem annotation ($n = 102$ subsystems), subsystem significance based on permutation test. **h,i** SEED subsystem annotation ($n = 81$ subsystems), subsystem significance based on Wilcoxon signed rank test. **d-i** Green symbols: mean $\pm$ s.d. (error bars); orange symbols: outliers; filled symbols: significant subsystems (identified below panels); solid black line: linear regression; dashed black lines: 90% confidence intervals for prediction by regression. See **Methods** for details.

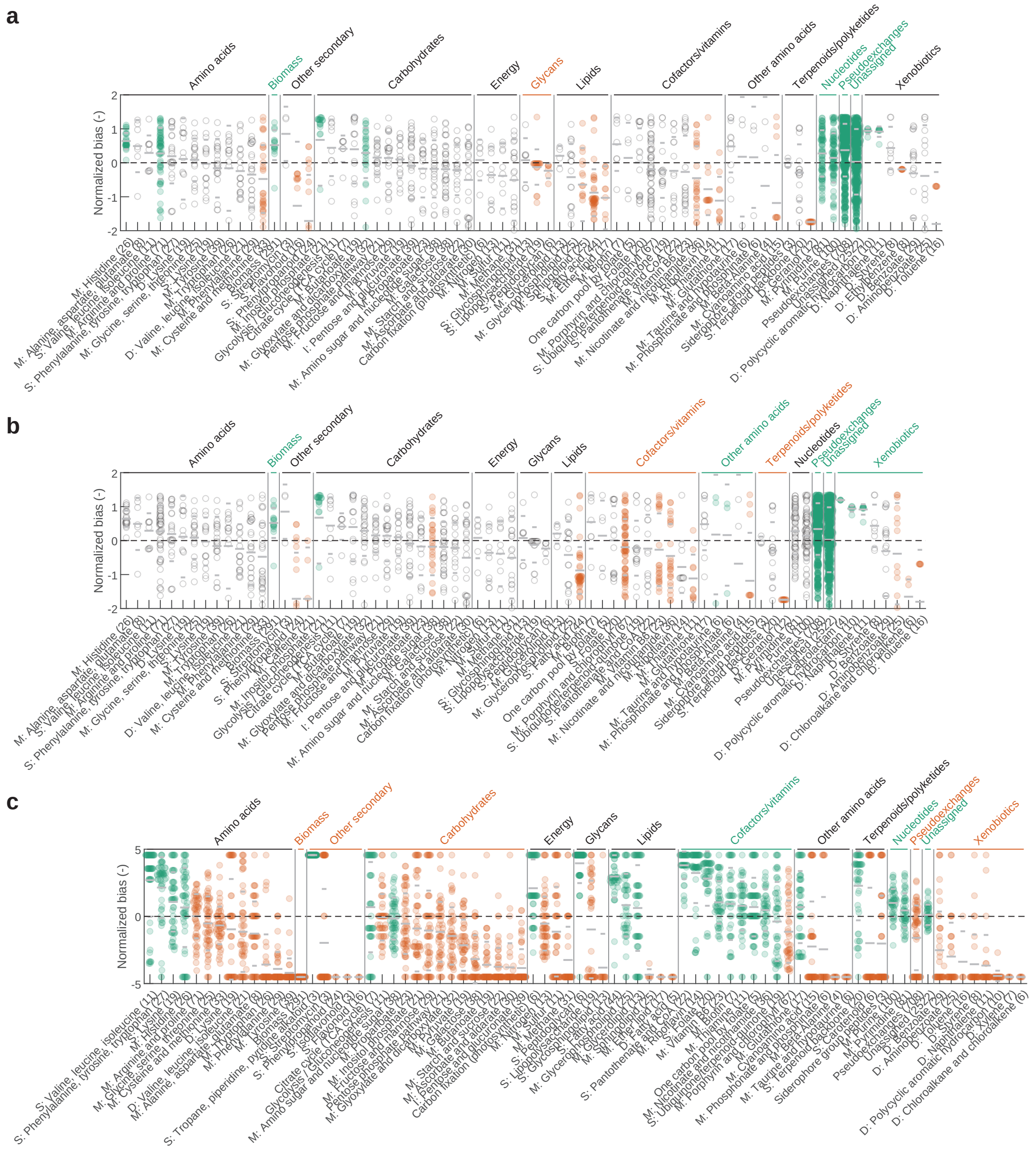

Figure S6. Alternatives for subsystem classification. a As **Fig. 4a** of the main text, but different comparison group for the test statistic. Subsystem classification based on Wilcoxon signed rank test for mean reaction sensitivities (Pearson correlation) compared to all reactions with assigned subsystems, KEGG subsystem annotation. **b** As (**a**) but subsystem classification based on permutation test compared to all reactions. **c** Subsystem classification based on Wilcoxon signed rank test for Jaccard indices for all reactions in each subsystem, compared to Jaccard indices for all reactions, KEGG subsystem annotation.

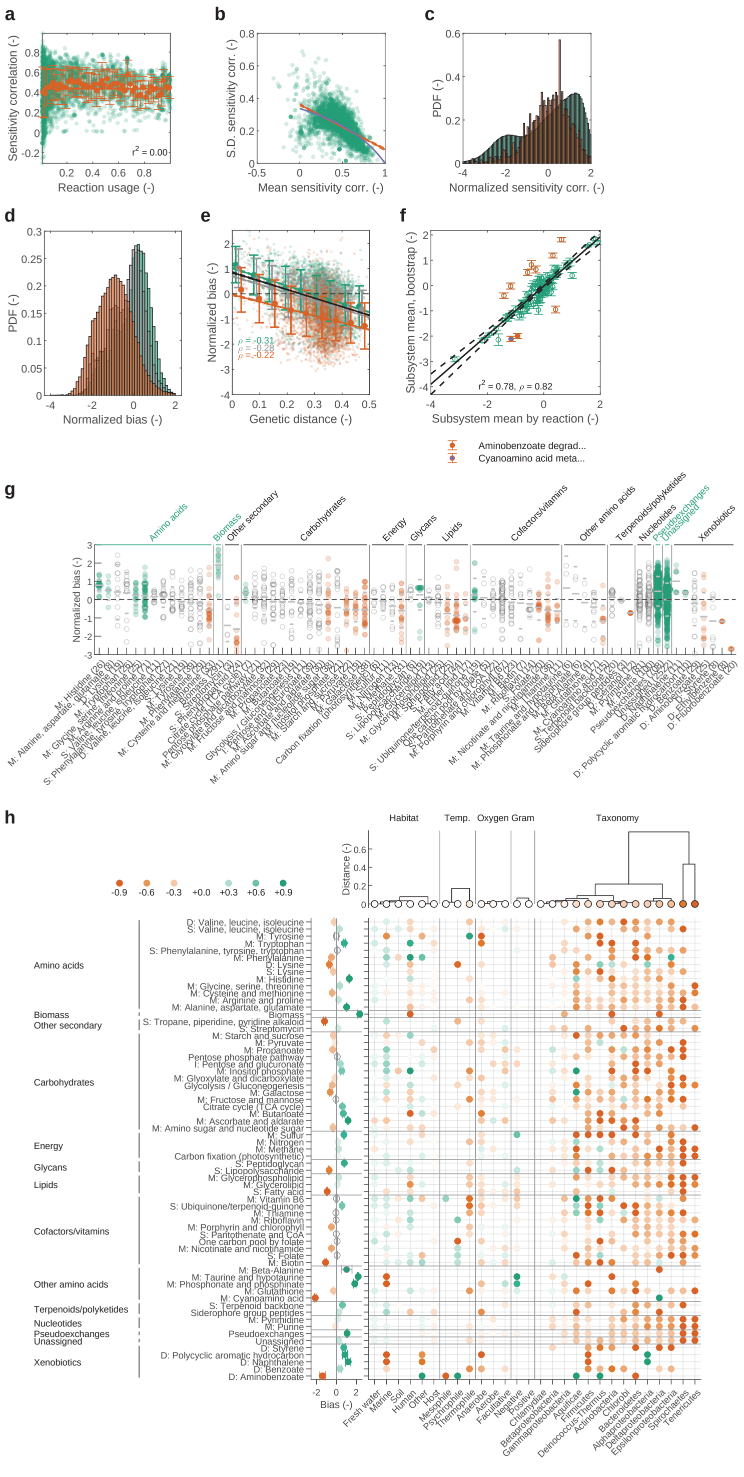

Figure S7. Sensitivity correlations computed via copulas instead of Pearson correlation. a Normalization of sensitivity correlations by linear regression as in **Fig. S4c** (average sensitivity correlations for $n = 3,491$ unique reactions). **b** Heteroskedacity of sensitivity correlations as in **Fig. S4d**. **c** Distributions of normalized sensitivity correlations as in **Fig. S4e**. **d** Distributions of normalized biases per reaction as in **Fig. S5a**. **e** Normalized bias per reaction classified by subsystem type as a function of genetic distance as in **Fig. S5c**; conserved (green), variable (orange) and other (gray) subsystems; small dots: individual data points ($n = 29,483$ per class); large dots and error bars (mean ± s.d.) of data binned by genetic distance; lines: linear regressions on individual data per class (colors) or for all classes (black).dots. **f** Correlations between average normalized biases per reaction and averages obtained by bootstrap of alignments using $n = 100$ samples as in **Fig. S5d** ($n = 102$ subsystems). **g** Subsystem classification based on Wilcoxon signed rank test for mean reaction sensitivities compared to all reactions, KEGG subsystem annotation, as in **Fig. 4a** of the main text. Only significant subsystems and those with at least one significant association in (**h**) are shown. **h** Functional variability depending on NCBI taxonomy, habitat, and physiology classes as in **Fig. 4b** of the main text.

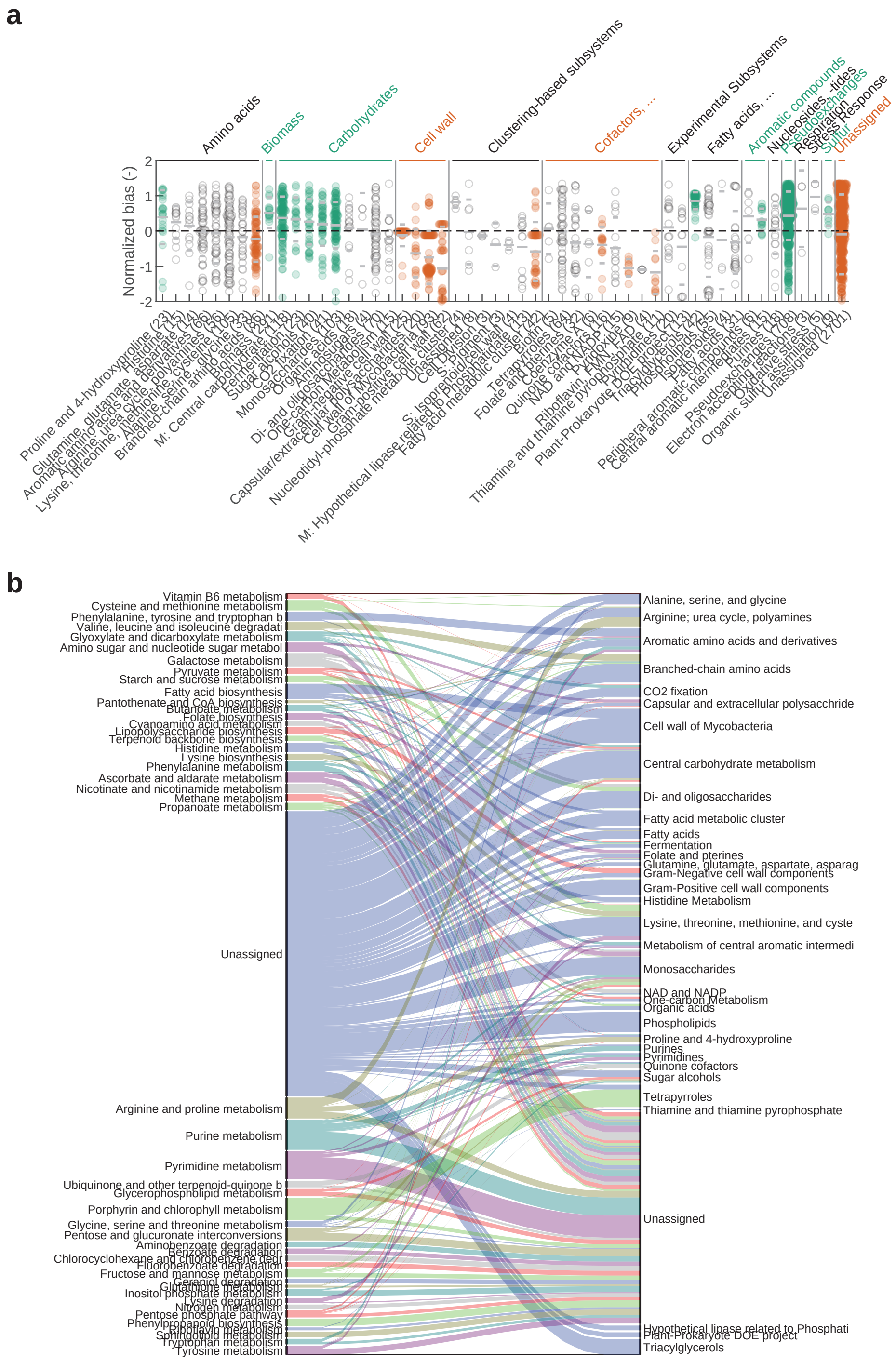

Figure S8. SEED subsystem annotations for reactions. **a** Subsystem classification as **Fig. 4a** of the main text, but with SEED subsystem annotation. **b** Flow diagram for mappings from re-annotated KEGG subsystems (left) to SEED subsystem annotation (right). Widths of flow lines correspond to numbers of reactions. Mappings between unassigned reactions are not shown.

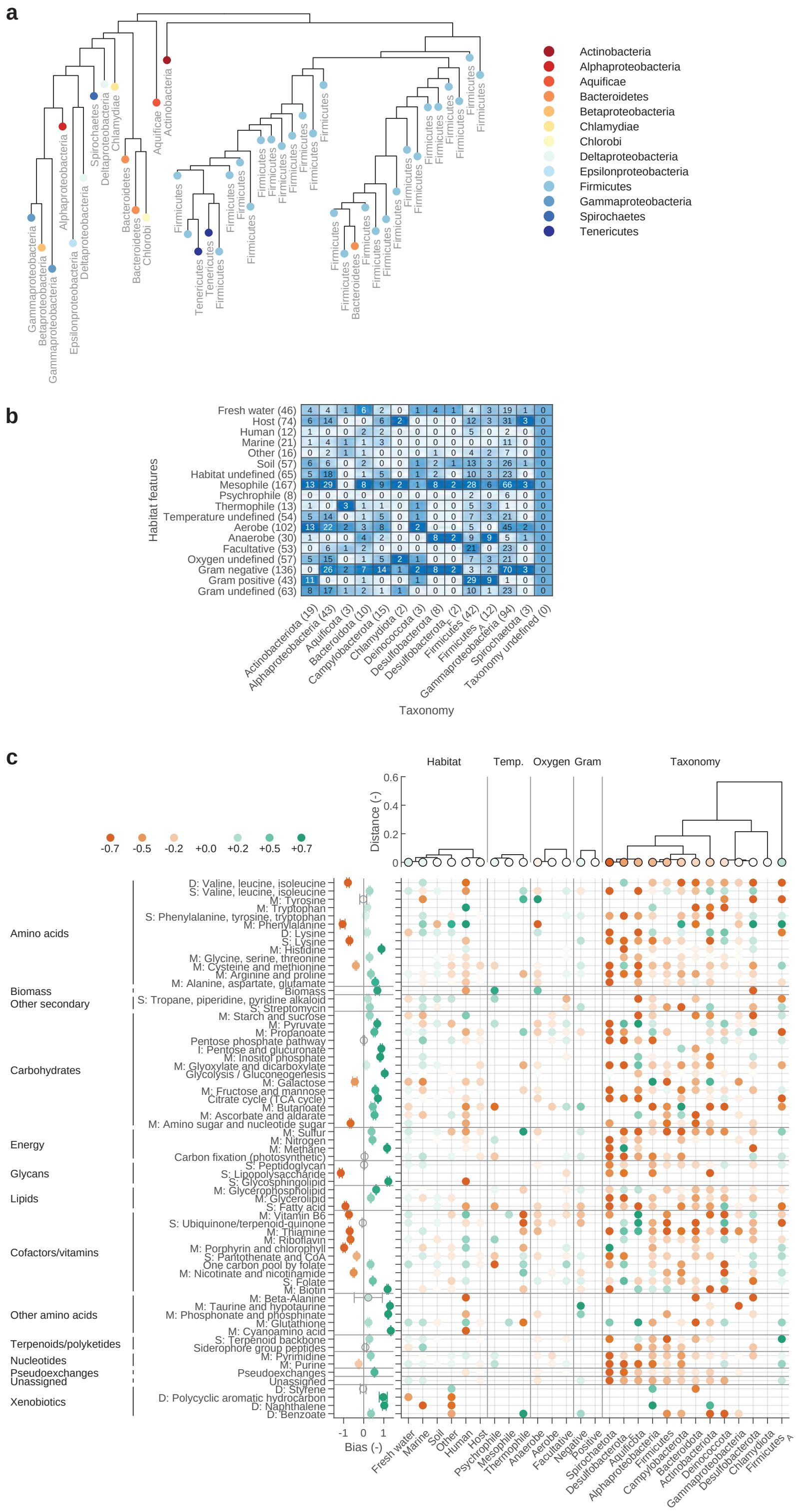

Figure S9. Subsystem analysis with phylogenetically consistent taxonomy. a Mapping of NCBI taxonomy to phylogenetic tree of bacteria[1]. Leafs contain species in the SEED GSM set with common assignments of taxa. **b** Model features as in **Fig. S4a** after taxonomic classification using the phylogenetically consistent taxonomy provided by the Genome Taxonomy Database (GTDB)[2,3]. **c** Functional variability as in **Fig. 4b** of the main text (n = 102 subsystems), but using GTDB taxonomy.

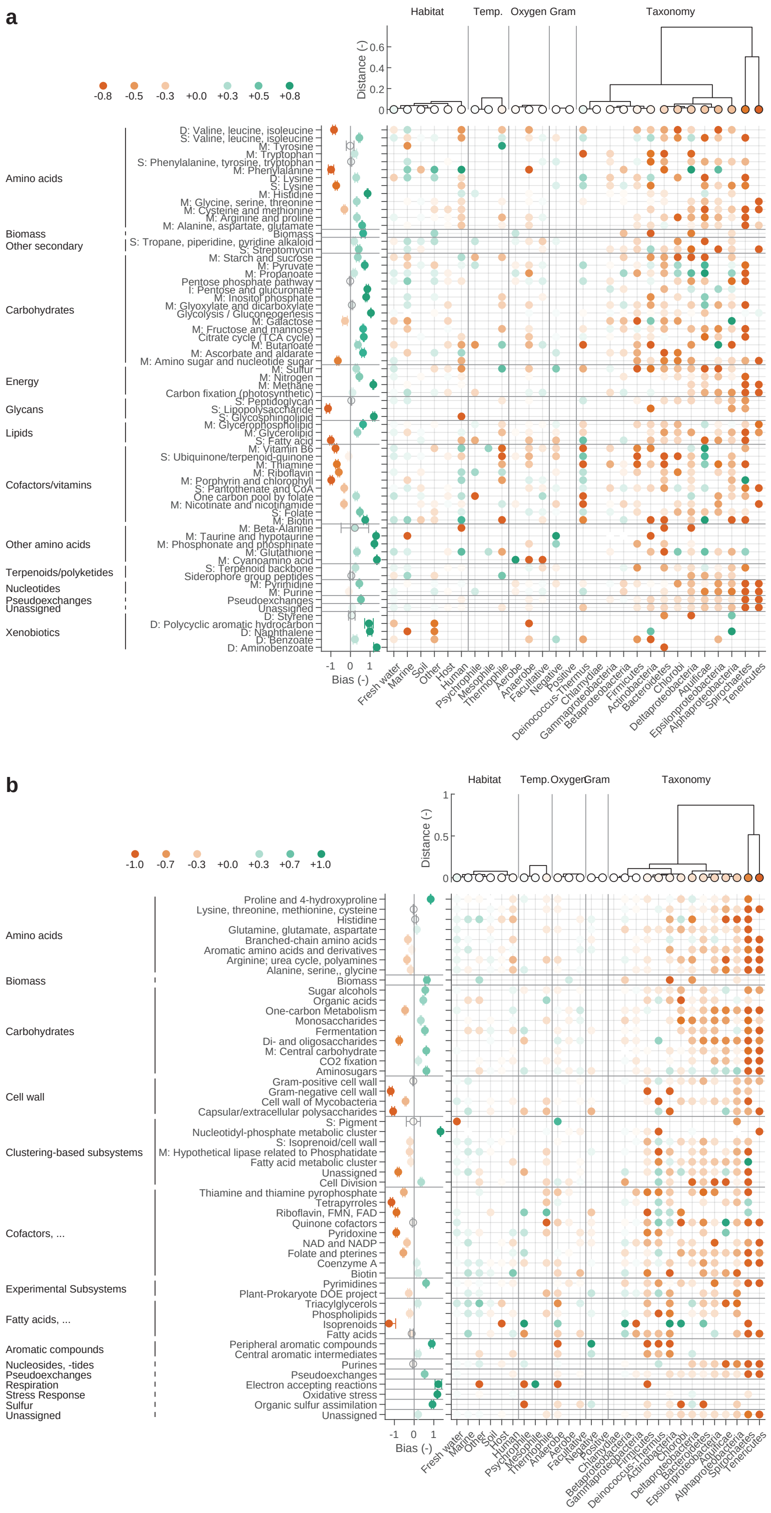

Figure S10. Alternatives for functional variability depending on NCBI taxonomy, habitat, and physiology classes. Functional variability as in **Fig. 4b** of the main text, but without considering unassigned reactions for linear regressions (**a**; $n = 102$ subsystems) and with SEED subsystem annotation (**b**; $n = 81$ subsystems), respectively.

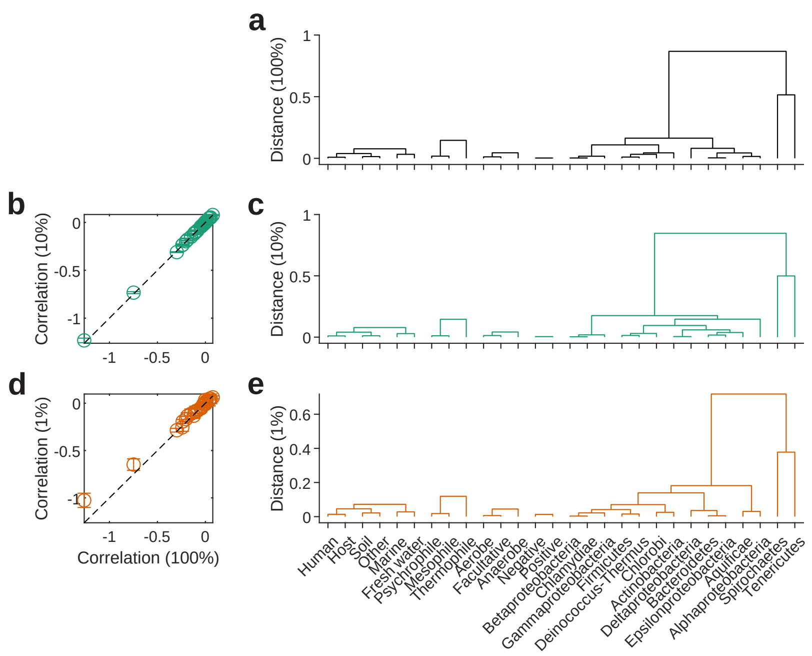

Figure S11. Robustness of clustering according to THP classes. a Reference tree based on regression coefficients for the full data set of sensitivity correlations (indicated by '100%') as in **Fig. 4b** of the main text. **b,d** Comparison of estimated regression coefficients for $n = 20$ bootstrap samples of sensitivity correlations per fraction (10% of full data set in **b**, 1% in **d** per sample; mean $\pm$ s.d.). **c,e** Hierarchical clustering based on average regression coefficients of bootstrap samples in **b,d**.

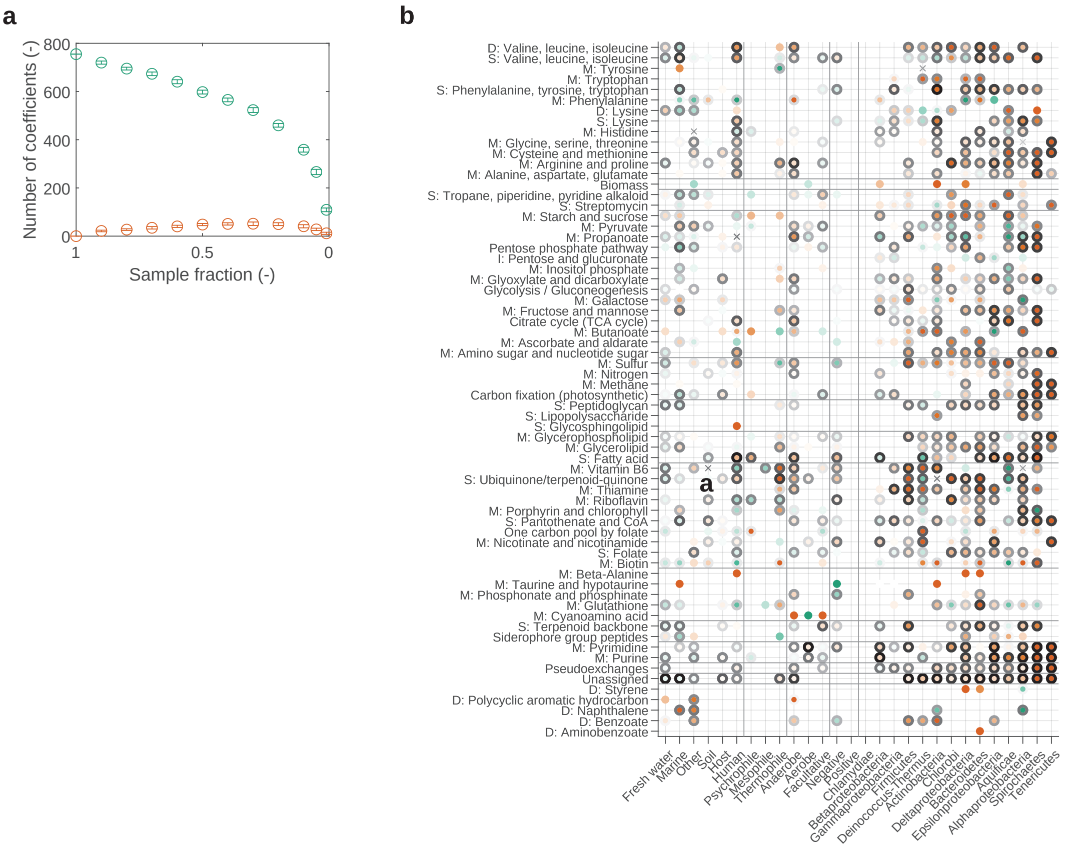

Figure S12. Robustness of hypotheses on subsystem conservation and variability. **a** Number of significant regression coefficients for subsamples of sensitivity correlations of the indicated fractions of the complete data set ($n = 20$ subsamples per fraction, mean $\pm$ s.d.). Green: coefficients were significant in all subsamples and of the same sign as in the full data set (true positives); orange: significant coefficients in all samples that were not significant in the full data set (false positives). **b** Summary of regression coefficients. Colored nodes as in **Fig. 4b** of the main text. Rings around nodes indicate robustness of hypotheses from light gray (true positive in 90% sample fraction) to black (true positive in 5% sample fraction). Crosses denote false positives.

Supplementary References

1. L. A. Hug, B. J. Baker, K. Anantharaman, C. T. Brown, A. J. Probst, C. J. Castelle, C. N. Butterfield, A. W. Hernsdorf, Y. Amano, K. Ise, Y. Suzuki, N. Dudek, D. A. Relman, K. M. Finstad, R. Amundson, B. C. Thomas, and J. F. Banfield. A new view of the tree of life. *Nature microbiology*, 1:16048, Apr. 2016. ISSN 2058-5276. doi: 10.1038/nmicrobiol.2016.48.

2. D. H. Parks, M. Chuvochina, D. W. Waite, C. Rinke, A. Skarshewski, P.-A. Chaumeil, and P. Hugenholtz. A standardized bacterial taxonomy based on genome phylogeny substantially revises the tree of life. *Nature biotechnology*, 36:996–1004, Nov. 2018. ISSN 1546-1696. doi: 10.1038/nbt.4229.

3. D. H. Parks, M. Chuvochina, P.-A. Chaumeil, C. Rinke, A. J. Mussig, and P. Hugenholtz. A complete domain-to-species taxonomy for bacteria and archaea. *Nature biotechnology*, 38:1079–1086, Sept. 2020. ISSN 1546-1696. doi: 10.1038/s41587-020-0501-8.
