# Supplementary material for: Functional comparison of metabolic networks across species
Source: Nat Commun. 2023 Mar 27;14:1699. doi: 10.1038/s41467-023-37429-5 (PMC10043025; doi:10.1038/s41467-023-37429-5)
Supplement: Supplementary file 3 — Description of Additional Supplementary Files [file 41467_2023_37429_MOESM3_ESM.pdf]

Description of Additional Supplementary Files:

Supplementary Dataset 1: Summary of SEED models (identifiers, main features, and taxonomy / habitat /physiology annotations). Columns whose identifiers start with ’has’ specify if a feature is defined, columns starting with ’is’ give the binary status of the feature. Taxonomy annotations in this context refer to NCBI taxonomy. Columns ’HabitatPreferenceOriginal’ and ’HabitatPreference’ contain original fusionDB entries and condensed habitat assignments, respectively.

Supplementary Dataset 2: Unified reaction annotations (KEGG and SEED classifications) for SEED models.

Supplementary Dataset 3: Detailed results (coefficients and statistical test results) for the analysis of functional variability of microbial metabolism depending on NCBI taxonomy, habitat, and physiology (THP) classes (Fig. 4b).
