# Supplementary material for: Functional comparison of metabolic networks across species
Source: Nat Commun. 2023 Mar 27;14:1699. doi: 10.1038/s41467-023-37429-5 (PMC10043025; doi:10.1038/s41467-023-37429-5)
Supplement: Supplementary file 7 — Reporting Summary [file 41467_2023_37429_MOESM7_ESM.pdf]

nature portfolio

Corresponding author(s): Joerg Stelling

Last updated by author(s): Mar 1, 2023

Reporting Summary

Nature Portfolio wishes to improve the reproducibility of the work that we publish. This form provides structure for consistency and transparency in reporting. For further information on Nature Portfolio policies, see our Editorial Policies and the Editorial Policy Checklist.

Statistics

For all statistical analyses, confirm that the following items are present in the figure legend, table legend, main text, or Methods section.

n/a	Confirmed	
☐	☒	The exact sample size (*n*) for each experimental group/condition, given as a discrete number and unit of measurement
☒	☐	A statement on whether measurements were taken from distinct samples or whether the same sample was measured repeatedly
☐	☒	The statistical test(s) used AND whether they are one- or two-sided<br>*Only common tests should be described solely by name; describe more complex techniques in the Methods section.*
☐	☒	A description of all covariates tested
☐	☒	A description of any assumptions or corrections, such as tests of normality and adjustment for multiple comparisons
☐	☒	A full description of the statistical parameters including central tendency (e.g. means) or other basic estimates (e.g. regression coefficient) AND variation (e.g. standard deviation) or associated estimates of uncertainty (e.g. confidence intervals)
☐	☒	For null hypothesis testing, the test statistic (e.g. *F*, *t*, *r*) with confidence intervals, effect sizes, degrees of freedom and *P* value noted<br>*Give P values as exact values whenever suitable.*
☒	☐	For Bayesian analysis, information on the choice of priors and Markov chain Monte Carlo settings
☒	☐	For hierarchical and complex designs, identification of the appropriate level for tests and full reporting of outcomes
☐	☒	Estimates of effect sizes (e.g. Cohen's *d*, Pearson's *r*), indicating how they were calculated

Our web collection on statistics for biologists contains articles on many of the points above.

Software and code

Policy information about availability of computer code

Data collection	We did not use any specific software for data collection.
Data analysis	All data analysis was carried out using Matlab scripts, which are provided in the github repository. The link can be found in the readme file of the repository (https://doi.org/10.3929/ethz-b-000598615).<br>- Matlab R2019b (Mathworks)<br>- Gurobi Optimizer (v8.1.1).<br>- Cobra toolbox for Matlab (v3.1)<br>- Jonker-Volkenqnt Algorithm for Linear Assignment Problem V3.0 (https://www.mathworks.com/matlabcentral/fileexchange/26836-lapjv-jonker-volgenant-algorithm-for-linear-assignment-problem-v3-0), MATLAB Central File Exchange. Retrieved September 30, 2021.)<br>- alluvialFlow (Downloaded from: https://www.mathworks.com/matlabcentral/fileexchange/66746-alluvial-flow-diagram)<br>- colorBrewer (ColorBrewer: Attractive and Distinctive Colormaps (https://github.com/DrosteEffect/BrewerMap), GitHub. Retrieved September 30, 2021)<br>- Functions were adapted from this publication (removeTrivialFC_cr.m) Larhlimi, A., David, L., Selbig, J., & Bockmayr, A. (2012). F2C2: a fast tool for the computation of flux coupling in genome-scale metabolic networks. BMC bioinformatics, 13(1), 1-9.

For manuscripts utilizing custom algorithms or software that are central to the research but not yet described in published literature, software must be made available to editors and reviewers. We strongly encourage code deposition in a community repository (e.g. GitHub). See the Nature Portfolio guidelines for submitting code & software for further information.

Data

Policy information about availability of data

All manuscripts must include a data availability statement. This statement should provide the following information, where applicable:

- Accession codes, unique identifiers, or web links for publicly available datasets
- A description of any restrictions on data availability
- For clinical datasets or third party data, please ensure that the statement adheres to our policy

The datasets generated during and/or analyzed are available at doi : https://doi.org/10.3929/ethz-b-000598615. All the data for reproducing figures in the main text and the supplementary information are provided in the GitHub repository associated. We used the following publicly available datasets:
1. Orthologues: retrieved using the OMA database, available at https://omabrowser.org/cgi-bin/gateway.pl?f=PairwiseOrthologs&p1=HUMAN&p2=YEAST&p3=EntrezGene
2. iMM904, iJO844, iJO1366, and Recon1 models were downloaded from Bigg (http://bigg.ucsd.edu/).
3. MetaNetX models were retrieved from MetaNetX (https://www.metanetx.org/).
4. SEED models were downloaded from the publication: Plata, G., Henry, C. & Vitkup, D. Long-term phenotypic evolution of bacteria. Nature 517, 369–372 (2015) (http://vitkuplab.c2b2.columbia.edu/phenotypes/)
5. NCBI taxonomy, available at https://www.ncbi.nlm.nih.gov/taxonomy
6. GTDB taxonomy available at https://data.gtdb.ecogenomic.org/releases/latest/
7. Species synonyms (MACADAM database), available at http://macadam.toulouse.inra.fr/doc/MACADAMDatabase.zip
8. Habitat and physiology annotation (FusionDB), available at https://services.bromberglab.org/fusiondb/explore
9. Gram status (Microbe directory), available at https://github.com/microbe-directory/microbe-directory/blob/master/data/microbe-directory.csv.
10. Model SEED annotations and reaction aliases, available at https://github.com/ModelSEED/ModelSEEDDatabase/blob/master/Biochemistry/

Human research participants

Policy information about studies involving human research participants and Sex and Gender in Research.

Reporting on sex and gender	There was no human subject in this study. Not applicable.
Population characteristics	There was no human subject in this study. Not applicable.
Recruitment	There was no human subject in this study. Not applicable.
Ethics oversight	There was no human subject in this study. Not applicable.

Note that full information on the approval of the study protocol must also be provided in the manuscript.

Field-specific reporting

Please select the one below that is the best fit for your research. If you are not sure, read the appropriate sections before making your selection.

☐ Life sciences ☐ Behavioural & social sciences ☒ Ecological, evolutionary & environmental sciences

For a reference copy of the document with all sections, see nature.com/documents/nr-reporting-summary-flat.pdf

Ecological, evolutionary & environmental sciences study design

All studies must disclose on these points even when the disclosure is negative.

Study description	We developed a method to compare perturbation phenotypes of metabolic reactions within metabolic networks in silico. This method gives a similarity score to a pair of metabolic reactions belonging to a pair of models. We then performed an observational study, where we compared our score to other similarity scores, such as EC number similarity score and other variables such as the properties of the organisms studied (i.e., temperature tolerance, habitat and phylogeny). Therefore no treatment factor or specific experimental design could be applied.
Research sample	We applied our method to various models obtained from various repositories (see above on data). The research samples are the sensitivity correlations of common reactions between pairs of genome -cale metabolic models, i.e. the similarity score. Each sample is meant to represent the similarity in the response to a flux perturbation of the reaction in each model.
Sampling strategy	Sampling was performed in the assignment problem used to align the yeast model with itself; we sampled randomly reactions in the copy model to check how the assignment performs. Then, in multiple instances in the manuscript, we check the robustness of our predictions by performing bootstrapping. This is the case in figure S2e, S5d, S11 and S12, where the sample size is the number of bootstrap samples used, which is in general n=100 except for S11 and S12 where a reduced number of bootstraps was used for computational reasons (n=20; variance of the estimator of the mean was sufficiently small for this sample size).

Data collection	We used existing published data as described in the Data section. We did not collect any data ourselves.
Timing and spatial scale	The timing and spatial scale is not relevant here since we studied in silico model organisms at steady-state.
Data exclusions	We excluded 76 models from the 321 seed models (removal of duplicate species and requirement of minimal number of species per taxonomic unit analyzed; see Methods for details).
Reproducibility	This study does not feature experimental findings. Yet, we used different classification schemes for subsystems. A discussion on the impact of using a different classification scheme is included in the manuscript. All analyses can be reproduced with the software contained in the git repository.
Randomization	Randomization was performed in the assignment problem used to align the yeast model with itself. We randomized the order of reactions in the copy and we randomly removed a fraction of the reactions in the copy. In other sections, the group allocations were performed based on fixed characteristics of the organisms (e.g. phylogeny), i.e., no treatment could be randomized on the species. Therefore, we validated some of our findings by performing bootstrapping ,i.e., we randomly sampled a fraction of the original dataset to validate the robustness of the findings.
Blinding	Blinding is only relevant in the assignment problem used to align the yeast model with itself. We blinded the name of reactions in order to perform the assignment. The assignment was only performed based on the calculated sensitivities and not the name of the reactions. In other sections, the group allocation was performed based on fixed characteristics of the organisms as above.

Did the study involve field work? ☐ Yes ☒ No

Reporting for specific materials, systems and methods

We require information from authors about some types of materials, experimental systems and methods used in many studies. Here, indicate whether each material, system or method listed is relevant to your study. If you are not sure if a list item applies to your research, read the appropriate section before selecting a response.

Materials & experimental systems

n/a	Involved in the study
☒	☐ Antibodies
☒	☐ Eukaryotic cell lines
☒	☐ Palaeontology and archaeology
☒	☐ Animals and other organisms
☒	☐ Clinical data
☒	☐ Dual use research of concern

Methods

n/a	Involved in the study
☒	☐ ChIP-seq
☒	☐ Flow cytometry
☒	☐ MRI-based neuroimaging
